# Adaptive demand forecasting framework with weighted ensemble of regression and machine learning models along life cycle variability

**DOI:** 10.1038/s41598-025-23352-w

**Published:** 2025-11-04

**Authors:** Islam M. Hammam, Amin K. El-Kharbotly, Yomna M. Sadek

**Affiliations:** Design and Production Engineering Department, Ain Shams University, Cairo, Egypt

**Keywords:** Mixed data patterns, XGBoost algorithm, Ensemble model, Product life cycle, Operations management, Mechanical engineering, Statistics

## Abstract

Accurate demand forecasting is essential for informed decision-making in today’s dynamic business environment, where product demand often follows diverse and shifting patterns throughout increasingly shorter life cycles driven by continuous product innovation. This study aims to develop a forecasting framework capable of accurately predicting demand across varying patterns, with particular attention to the decline phase of the product life cycle. Traditional statistical forecasting methods, such as those in the ARIMA family, generally perform well with linear trends over short horizons, whereas machine learning techniques like XGBoost are better suited for capturing complex, nonlinear patterns over longer periods. This paper introduces an adaptive, hybrid forecasting framework that integrates ARIMA-based regression models with XGBoost using a weighted ensemble strategy. Initially, the framework tests linear models; if diagnostic analysis indicates nonlinearity, it incorporates XGBoost to address these complexities. To optimize the ensemble model performance, a grid search algorithm adjusts the ensemble weights by minimizing the root mean square error (RMSE), enabling the framework to dynamically leverage the strengths of both approaches. The proposed method was validated on five datasets representing different phases of the product life cycle. Results demonstrate that the proposed framework achieved MAPE below 13% on most datasets, with up to 80% improvement over ARIMA models in cases involving high variability demand patterns. The results show that the ensemble model enhances both flexibility and accuracy, especially for demand patterns that combine linear and nonlinear components. The framework benefits from the explainability and time-series capabilities of ARIMA while utilizing XGBoost’s power to model nonlinear relationships. This research underscores the practical advantages of hybrid modeling in improving demand forecasting and operational planning across various industry sectors.

## Introduction

Demand forecasting is the process of accurately estimating the demand for a product by considering various independent input variables and their relationship with the demand. It is the cornerstone of supply chain management and prediction of product life cycle. Many factors that affect the demand are random, uncertain, fuzzy, and have a nonlinear relation with the demand. This makes it challenging to establish precise mathematical models^1^. For decades, time series forecasting has been studied across different fields such as statistics, econometrics, mathematics, engineering, etc. Despite their good results in forecasting, novel statistical methods (like ARIMA family) are limited to the scope of linear and near linear assumptions^2^. Artificial intelligence can develop algorithms that can improve performance by experience. Machine learning as a subfield of artificial intelligence can make decisions, predictions and forecasting based on historical data without the limitations of linear assumptions^3^. Machine learning does not need to be programmed explicitly for a certain task^4^. Instead, it provides an effective solution where traditional approaches may fall short, allowing prediction or decision-making based solely on data-driven information.

Research on forecasting methods has been conducted to utilize both regression models and machine learning algorithms, comparing their performance with common benchmark models such as Autoregression Integrated Moving Average (ARIMA). For instance, Villegas et al.^5^ employed Support Vector Machine (SVM) to choose the most suitable prediction model from several predictive models for scenarios that involve unstable demand in a short period. Ji et al.^6^ introduced a three-stage hybrid forecasting method based on Clustering, Extreme Gradient Boosting (XGBoost), and ARIMA which was tested against multi-featured e-commerce datasets along with other models showing exceptional performance compared to traditional and machine learning methodologies. Pin Li and Jin-Suo Zhang^7^ developed a hybrid model that combines ARIMA with XGBoost to forecast China’s energy supply security. They compared the accuracy of their ARIMA-XGBoost hybrid model against an ARIMA-only approach based on mean absolute percentage error (MAPE) results, which were lower than 4.5%. As a result, they concluded that the hybrid model was more precise and closer to actual outcomes. Yan Wang and Yuankai Guo^8^ decomposed the stock historical data set using discrete wavelet transform (DWT) into—a partial data set and an error-related dataset—with the use of the grid search algorithm to optimize the XGBoost parameters and construct the grid search XGB (GSXGB) model. Among all candidate models of ARIMA, XGBoost, GSXGB, DWT-ARIMA-XGBoost and DWT-ARIMA-GSXGB, the last one showed better accuracy and generalization ability according to the simulation results.

A data-driven analytics framework was developed by Wenhan Fu and Chen-Fu Chien^9^ for predicting the demands of intermittent electronic components. To counteract discontinuous demand patterns, temporal aggregation and a combination forecast using Syntetos-Boylan approximation, ARIMA, and Recurrent Neural Network (RNN) were employed. The findings indicated that this integrated approach with temporal aggregation can effectively facilitate flexible decision-making to support supply chain innovation in electronics. Similarly, Ping Jiang and Ranran Li^10^ proposed a composite model for forecasting electricity demand. Their modeling concept exhibited an impressive ability to detect seasonal relationships within electricity demand data as well as superior performance accuracy compared to benchmark models. Yanzhi Duan and Sensheng Li^11^ tested the result of XGBoost algorithm for forecasting short term urban gas daily demand against other machine learning models considering some features affecting the demand. The XGBoost returned excellent results against multi regression, random forest, and Support Vector Machine (SVM). Wang et al.^12^ proposed a hybrid forecasting model combining ARIMA and LSTM to improve short-term demand prediction for IC trays in the semiconductor industry. The study addresses highly volatile and intermittent demand patterns under short lead-time and on-time delivery constraints. The performance of ARIMA and LSTM was evaluated using MAPE and RMSE, with results showing that LSTM significantly outperformed ARIMA and the company’s empirical forecasting method. The study highlights the superior performance of LSTM in handling nonlinearities and short-term dynamics, while also acknowledging the strengths of ARIMA in modeling linear trends. İmece and Beyca^13^ developed an ensemble model for the pharmaceutical industry by integrating time series and regression methods. Using actual daily sales data and features like promotions, holidays, price changes, and stock availability, the authors tested Holt-Winters, Ridge Regression, Random Forest, and XGBoost models. Their ensemble strategy—based on inverse-error weighting—achieved the lowest RMSPE (10.7%) with a Holt-Winters & XGBoost combination, outperforming all individual models. The study confirmed that hybrid ensembles enhance forecasting accuracy by capturing both temporal and input-driven demand variations. Aswanuwath^14^ proposed a hybrid model for forecasting daily electricity peak loads using variational mode decomposition (VMD) and fast Fourier transform (FFT) to extract seasonal components. The model incorporated stepwise regression and similar-day selection for input variable optimization, leading to improved forecast accuracy and reduced computational complexity with minimizing neural network structure requirements as well.

Ozdemir and Yozgatligil^15^ conducted a comprehensive comparison of machine learning, statistical, and hybrid models across various time domains. Their results showed that machine learning models, such as XGBoost and LSTM, generally outperformed traditional statistical methods for complex time series. However, the study emphasized that hybrid models do not always yield superior results, as performance depends heavily on the characteristics of the underlying data. Çaglayan-Akay and Topal^16^ compared traditional models (e.g., SARIMA) with hybrid approaches for forecasting electricity consumption in Türkiye. Their results showed that the Khashei & Bijari hybrid model outperformed both single and alternative hybrid models, effectively capturing the nonlinear structure of the data and confirming the advantage of hybrid methods over traditional linear models. Duan and Dong^17^ proposed an ensemble demand forecasting model tailored for the home appliance industry, combining LSTM, Random Forest (RF), and XGBoost within a two-layer blending framework. The first layer consisted of base learners trained on distinct features, while the second layer used a Multiple Linear Regression (MLR) meta-learner to optimize predictions. Applied to a large-scale dataset of air conditioner sales in China, the hybrid model achieved superior accuracy (R^2^ = 0.9116) compared to single models, demonstrating enhanced performance in capturing complex demand dynamics driven by seasonal, promotional, and behavioral factors.

From the previous literature review, it is found that recent studies have explored a wide range of forecasting techniques, including classical models such as ARIMA and SARIMA, as well as machine learning methods like Support Vector Machines, Random Forests, and XGBoost. Some hybrid models have been proposed to improve accuracy by combining statistical and AI-based approaches. However, these models often focused on either linear or nonlinear patterns in isolation, and few provided a data-driven mechanism to adapt dynamically based on the structure of the time series. Moreover, there is limited focus in the literature on forecasting during the declining phase of the product life cycle, which poses unique challenges due to volatility and structural changes in demand. To address these limitations, this study proposes a novel, adaptive forecasting framework that integrates statistical regression models and machine learning (XGBoost) using a Weighted Average Ensemble (WAE) approach. The framework is utilized to forecast demand along different phases of the product life cycle as an important application, focusing on the stage of decline as the main challenge among the other phases. The regression model addresses linear patterns in the data, while the machine learning model addresses non-linear demand pattern^18^. Additionally, the proposed framework integrates the outcomes of the regression model and the machine learning techniques through a weighted average ensemble (WAE) algorithm to benefit from the strengths of both models for mixed patterns. This framework will help forecast different phases of the product life cycle. The performance of the models will be evaluated using the most popular metrics; mean absolute error (MAE), mean square error (MSE), root mean square error (RMSE), and mean absolute percentage error (MAPE). The main contribution of this work lies in providing a flexible, interpretable, and performance-optimized forecasting framework capable of handling mixed demand patterns, with particular attention to the underexplored declining phase. This framework aims to support more accurate and data-responsive demand planning in real-world industrial applications.

The paper is outlined as follows: “The developed forecasting framework” section presents the developed forecasting framework, including data preparation, model development, and the ensemble integration strategy. “Computational results and discussion” section details the experimental setup, datasets, and model evaluation metrics. “Conclusion and future work” section discusses computational results and compares the performance of different models across various demand patterns. “Data availability” section concludes the paper by summarizing the key findings and directions for future research.

## The developed forecasting framework

**Fig. 1:**
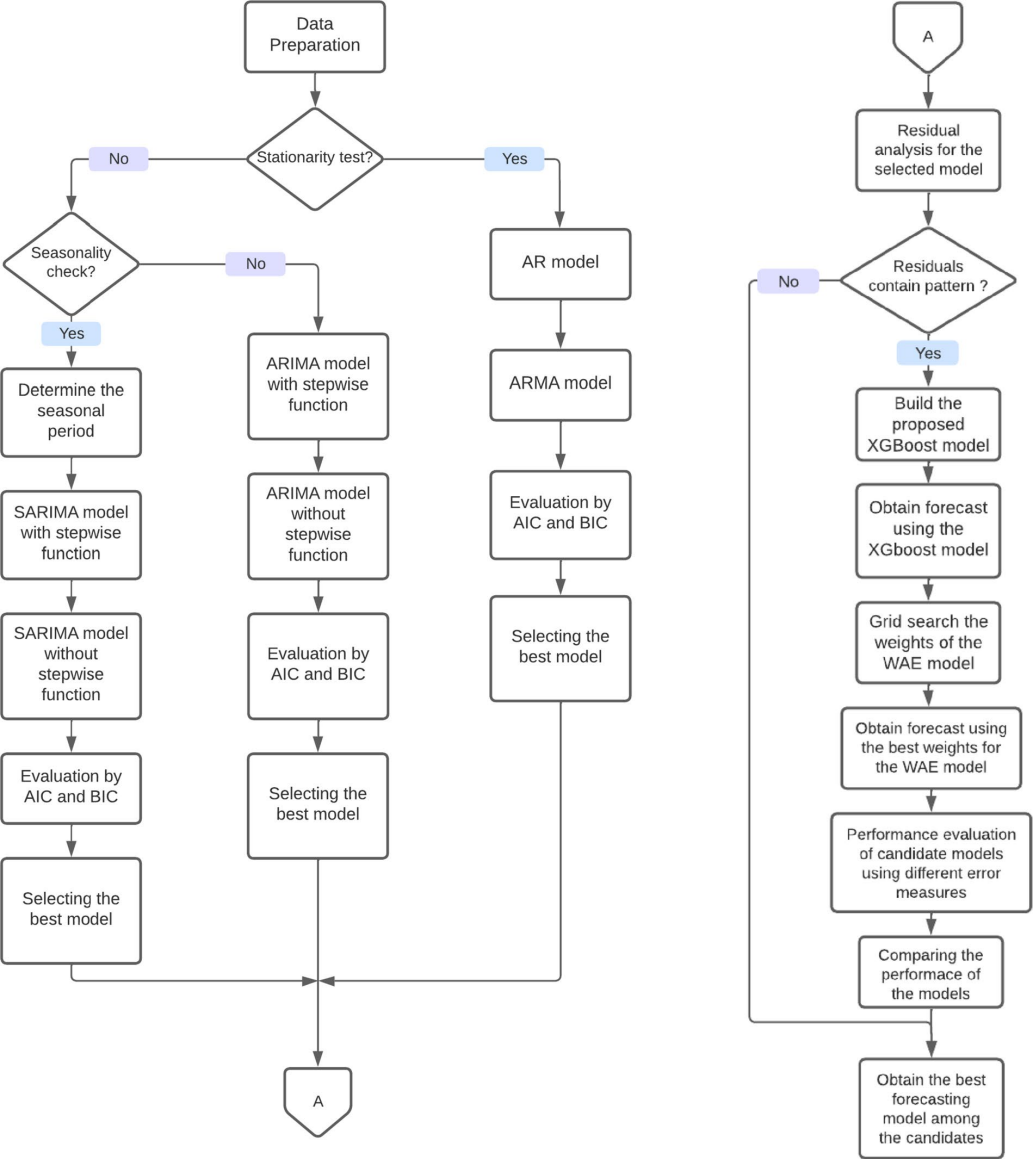
The developed framework.

The proposed framework commences by the assumption that the data to be forecast primarily follows a linear pattern. Then, data undergoes tests to grasp seasonal and stationary patterns. Subsequently, the framework determines the optimal regression model for generating forecasts. Once the forecasts are generated, the residuals are scrutinized to identify any patterns that the regression model may have missed. The absence of patterns validates the fundamental assumption of data linearity. If any patterns are identified in the data, the framework proceeds to create forecasts using XGBoost, followed by the development of the ensemble model to assess its performance against regression and XGBoost models. However, the framework has certain limitations. The framework is more suited for medium-to-large time-series datasets with smooth and erratic demand patterns. It was not recommended for very small-sized datasets—less than 100 observations—or those characterized by intermittent and lumpy demand patterns. This is because the XGBoost model is typically more data-hungry and may not perform optimally with very small datasets^19^. The framework returns the forecast of the best model based on the error measures. The proposed framework is detailed below and is depicted in Fig. 1.

### Data preparation

Generally, this step ensures that the subsequent modelling processes can focus on identifying meaningful patterns rather than correcting data issues, leading to more robust and actionable insights^20^. Main steps of data preparation are:

- Data Cleaning: Identifying and removing outliers or anomalies to reduce noise. Specifically, any data point beyond ± 3 standard deviations from the rolling mean was excluded
- Handling Missing Values: Filling or removing missing entries to maintain dataset consistency using the dropna() method.
- Formatting: Organizing data into a time-series format with equally spaced intervals for accurate modelling.
- Indexing: Ensuring the dataset is appropriately indexed to facilitate time-based analysis and forecasting.

Subsequently, two tests are conducted to identify stationarity and seasonality demand patterns. This step is pivotal for making the right model selection in the subsequent step of the framework. Stationarity tests assess if a time series data’s statistical properties remain constant over time. This framework uses two of the most common methods; Augmented Dickey Fuller test (ADF) and rolling statistics. In the ADF test, the p-value helps determine whether the data is stationary; if the p-value is below a critical threshold of 0.05, the null hypothesis of non-stationarity is rejected, indicating the data is stationary. Rolling statistics involve calculating a moving average or moving variance over a defined window of time, allowing for a visual assessment of stationarity by observing whether the mean and variance remain constant over time. When the results of the ADF test and rolling statistics yield conflicting interpretations, seasonal decomposition (SD) is used as an additional tool to validate the stationarity of the time series. The existence of trend and/or seasonal patterns indicates data non-stationarity. Using multiple tests increases robustness and reduces the risk of model misidentification.

Seasonality tests identify the recurring patterns or fluctuations that occur at regular intervals along the data set. These tests consider various methods; SD, autocorrelation function (ACF) and partial autocorrelation function (PACF). If the analysis proves seasonality in data, a periodogram is used to identify the seasonal period.

Afterwards, data is divided into sets. For the regression models, data is divided into two sets based on the common practice: training and testing sets in percentages 80% and 20% respectively^21^. For the XGBoost algorithm, the data is divided into training, validation, and testing sets in percentages 65%, 15% and 20% respectively to maintain the same size for the test data and have enough data for training process as well.

### The regression models

Regression models are statistical models that are used for predicting future values in time series by modeling the data’s underlying patterns, including autoregression, integration (differencing), and moving averages based on past observations^22^.

#### The regression models description

The ARIMA model stands out as one of the most widely used models for forecasting and is considered as a benchmark model in many studies^23,24^. The general form of the ARIMA model, including terms for autoregression, integration, and moving average components is shown in Eq. (1). Based on the tests conducted during the preparation phase, the framework selects the most appropriate regression model among. For stationary, non-seasonal data, both the autoregressive (AR) and autoregressive moving average (ARMA) models are utilized. For non-stationary, non-seasonal data, the autoregressive integrated moving average (ARIMA) model is chosen. Meanwhile, for non-stationary, seasonal data patterns, the seasonal autoregressive integrated moving average (SARIMA) model is selected. The explanation of the models and their parameter selection were detailed by Box et al.^22^. However, for ARIMA and SARIMA models, the model is constructed twice as M1 and M2: once with the assistance of the stepwise function and another without it. The results of both models are compared to select the model with the best performance. The performance is measured by the two widely used information criteria: Akaike Information Criterion (AIC) and Bayesian Information Criterion (BIC). Both metrics assess model quality by balancing goodness of fit with model complexity, where lower values indicate a more optimal trade-off between accuracy and simplicity. Hence, the regression model with minimum AIC and BIC is selected.

1 $$ARIMA (p,q,d): \varphi \left(B\right){\left(1-B\right)}^{d}{y}_{t}= \Theta \left(B\right){\varepsilon }_{t}$$

where: $\varphi \left(B\right)$ and $\Theta \left(B\right)$ are polynomials in the backshift operator $B$, $d$ is the order of differencing, and ${\varepsilon }_{t}$ is white noise.

#### Regression model diagnostics

This step is very crucial in the framework as it examines the residuals of the model to examine the initial hypothesis of data linearity to confirm or reject it. If the analysis of the residuals indicates a clear pattern, this is an indicator that the data has a nonlinear pattern. As the regression model managed to handle only the linear and seasonal patterns of the data – if existed-the framework utilizes the XGBoost algorithm to perform new forecasts. In this context, the “plot_diagnostics” function from the “statsmodels” library in Python is used to visualize the diagnostic plots for the fitted time series model. The four key diagnostic plots that “plot_diagnostics” generate are standardized residuals over time, histogram plus estimated density of standardized residuals, normal Q-Q plot, and correlogram. The four plots are widely accepted in the statistical forecasting literature^22,25^ as key tools for diagnosing the adequacy of linear models.

### The XGBoost forecasting model

XGBoost is an advanced machine learning technique based on the gradient boosting framework that was proposed by Chen and Carlos^19^ as an improved Gradient Boosting Decision Tree (GBDT). XGBoost is the selected machine learning technique in this study due to its high performance in forecasting nonlinear tabular datasets of various sizes and it consistently achieves state-of-the-art results^26^. We define the XGBoost model prediction as in Eq. (2). The proposed XGBoost model employs hyperparameters, with parameter optimization carried out through a grid search method, enhancing its overall performance. The model passes through five main steps:

2 $$\hat{y}_{t} = \mathop \sum \limits_{k = 1}^{k} f_{x} \left( {x_{t} } \right), f_{x} \varepsilon F$$

where each ${f}_{x}$ is a regression tree, $F$ is the space of CART models, and ${x}_{t}$ is the input feature vector at time $t$.

- *Features extraction*: Creating features using lag variables is a common technique employed in time series analysis to capture the temporal dependencies between past and future observations of a time series. The fundamental concept involves generating new features, which correspond to columns in a dataset, to represent the values of the time series at different time lags. After examining the ACF and PACF of the time series, significant lags were tested to create lagged features, also known as time lags or lag variables.
- *Splitting the data*: The data set is divided into train, validation, and test sets as explained in “Data preparation” section. The train set is utilized for model training and algorithm parameter tuning. Subsequently, the validation set is employed to assess the model’s performance based on the selected objective function, which in this case is RMSE. It also facilitates early stopping for the XGBoost model, enabling training to halt if the performance on the validation set begins to deteriorate. This serves to prevent overfitting and saves training time by avoiding unnecessary iterations.
- *Hyperparameters selection*: There are around 35 main hyperparameters for the XGBoost model. Those hyperparameters can be categorized into three types: General Parameters, Booster Parameters, Learning Task Parameters^27^. In this study six hyperparameters were selected. These are the most important and common ones^26^. Table 1 lists the hyperparameters.

**Table 1:** XGBoost hyperparameters.

Hyperparameter	Grid	Value	Comment
Learning rate	[0.01, 0.1]	0.1	Moderate value, gives relatively gradual learning, prevents overfitting
Maximum depth of the tree	[3–5]	5	Moderate depth gives complex patterns without overfitting
Number of trees	[100, 200, 300]	200	Moderate boosting rounds with low learning rate for better learning
Colsample_bytree	[0.8, 1]	0.8	Moderate value, increases robustness, reduces risk of overfitting
Reg_alpha	[0.001, 0.01]	0.001	Slight regularization, promots sparsity and preventing overfitting
Subsample	[0.6, 0.8]	0.6	Low to moderate value increasing robustness and randomness
  Limiting the model to only six core hyperparameters can help reducing the computional cost of the model to select the optimal values of the hyperparameters.
- *Setting hyperparameters grid*: This is accomplished using the “GridSearchCV” function from Python’s scikit-learn library from defined grid. This involves specifying a grid of values for each hyperparameter to reduce the tuning time. Table 1 column 2 shows the selected grids for each hyperparameter and the selected values for each parameter for the data set. It also shows the effect of the selected hyperparameter values on the model.
- *Hyperparameters tuning:* Using the defined hyperparameters and grids, the cross validation in this proposed framework is done through 3 folds. The optimal hyperparameters – shown in Table 1 – are selected based on their performance, with the minimum MSE as the evaluation metric.
- *Feature importance*: This is a method utilized to ascertain which features in a dataset exert the most significant impact on the target variable in a predictive modeling scenario. It aids in comprehending the relationship between features and the target variable. F-score (or frequency) is a straightforward measure used in this study for measuring the importance of the features to show how often a feature contributes to partitioning the data.

Once the hyperparameters of the model are set, the forecast is obtained.

### The weighted average ensemble (WAE) model

Beside the results of the regression model and the XGBoost algorithm, the WAE is used to give forecasts that combine both results. According to Brownlee^28^ “The weighted average or weighted sum ensemble is an extension over voting ensembles”. The regression model addresses linear patterns, while the XGBoost model addresses nonlinearity^18^. Hence, The development of the WAE using only two base learners to benefit the advantages of both. Using two models only helps the framework to not be computationally expensive. The forecasting results of the regression model and XGBoost model are assigned weights ${\omega }_{1}$ and ${\omega }_{2}$ respectively as in Eq. (3), ${\omega }_{1}$ and ${\omega }_{2}$ are determined through a grid search process with values ranging from 0 to 1 in increments of 0.1. The framework systematically explores the optimal combination of weights that minimize the root mean square error (RMSE). The mathematical formula for this model forecast can be expressed as:

3 $${F}_{WAE}= {\omega }_{1}{F}_{Regression}+ {\omega }_{2}{F}_{XGBoost}$$

where: $$\omega_{1} + \omega_{2} = 1, \omega_{1} , \omega_{2} \varepsilon \left\{ {0.0,0.1,...,1.0} \right\}$$

### Models evaluation

To measure the performance of the models used in this study, the test set of each dataset is compared to its forecast. The framework uses the most popular error metrics; MSE, RMSE, MAE, and MAPE. The best candidate model is the one that results in minimum error. These evaluation metrics are critical in comparing the predictive accuracy and consistency of the models across various datasets. In particular, the MSE, RMSE, MAE, and MAPE for series y at time series t is given in Eq. (4) - (7) respectively.

4 $$\text{MSE }=\frac{1}{T} \sum_{t=1}^{t}{\left({x}_{t}-{y}_{t}\right)}^{2}$$

5 $$\text{RMSE }= {\left(\frac{1}{T} \sum_{t=1}^{t}{\left({x}_{t}-{y}_{t}\right)}^{2}\right)}^{1/2}$$

6 $$\text{MAE }= \frac{1}{T} \sum_{t=1}^{t}\left|{x}_{t}-{y}_{t}\right|$$

7 $$\text{MAPE }=\frac{1}{T} \sum_{t=1}^{t}\left|\frac{{x}_{t}-{y}_{t}}{{x}_{t}}\right|*100$$

where: ${x}_{t}$ is the true value,

${y}_{t}$ is the predicted value,

T is number of observations.

## Computational results and discussion

The proposed framework was applied to forecast demand for five data sets. The datasets were selected to represent the different demand patterns for the different stages of the product life cycle curve. The conventional curve comprises four primary stages: introduction, growth, maturity, and decline. The focal points of this research are the challenging maturity and decline stages, characterized by shifts in the linear trend.

### Data sets under study

**Fig. 2:**
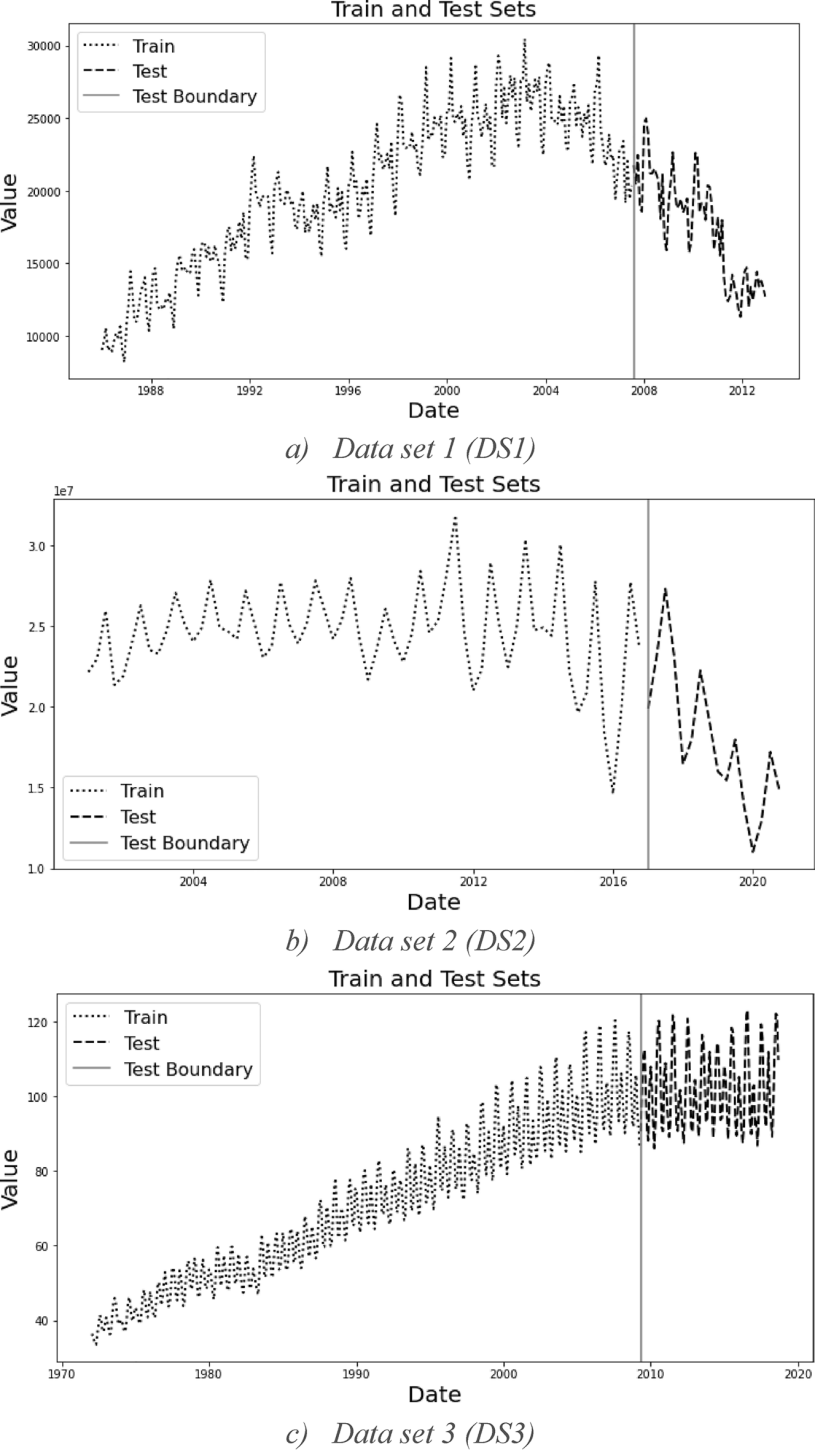

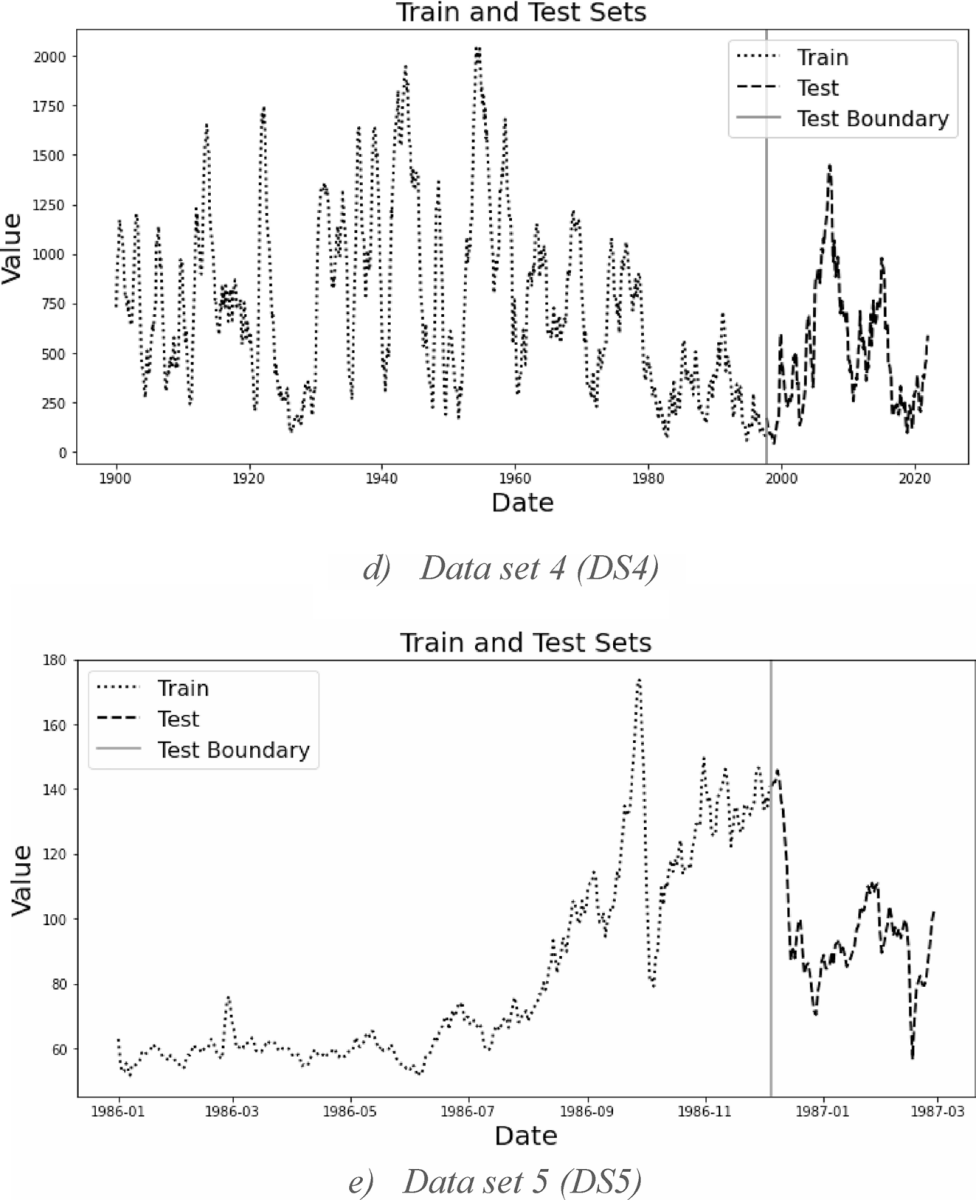
The five data sets under study are divided into train and test sets.

Five data sets (DS1, DS2, DS3, DS4, and DS5) are obtained from^29,30^. The data sets were selected for this study to represent three main demand patterns: linear, nonlinear, and mixed pattern (the pattern varies between linear and non-linear) as shown in Fig. 2. These datasets simulate three specific phases of the product life cycle:

- Phase1—Initial Demand: represents the introduction and the growth stages.
- Phase2—Saturated Demand: represents the maturity phase.
- Phase3—Diminishing Demand: represents the declining phase.

DS1 represents the entire PLC curve, with the test set is a part the diminishing demand phase—as the whole phase starts from the star mark shown in Fig. 2a. DS2 is a segment of the saturated demand, with a test set similar to DS1 case. DS3 captures the transition from the growth to the maturity stage, with the test set located in the saturated demand phase. DS4 exclusively covers the maturity phase, while DS5 focuses on the initial demand, with the test set in the initial demand phase.

Figure 2 shows the five datasets; each divided into a train and a test set in percentages 80% and 20%. Next, the stationarity test was conducted revealing that four sets; DS1, DS2, DS3, and DS5 are non-stationary, whereas DS4 are stationary. Next, the seasonality test was conducted on the non-stationary data sets to reveal that DS1, DS2, and DS3 have seasonal trends. According to the proposed framework, forecasting for the first three sets will take place using SARIMA, whereas DS4 will be forecasted using AR and ARMA. For DS5, the selection is the ARIMA model. These results are tabulated in Table 2.

**Table 2:** Data sets characteristics and tests results with regression model selection.

Data set	Frequency	Size	Demand type	*P* value	Stationary	Seasonal	Seasonal period	Model used
DS1	Monthly	324	Smooth	0.489	No	Yes	12	SARIMA
DS2	Monthly	80	Smooth	0.999	No	Yes	12	SARIMA
DS3	Monthly	560	Smooth	0.41	No	Yes	12	SARIMA
DS4	Monthly	1486	Erratic	3.5e-05	Yes	No	–	AR & ARMA
DS5	Monthly	423	Erratic	0.114	No	No	–	ARIMA

### Framework results for DS1

The results of applying the proposed framework on DS1 are presented and discussed in full detail. DS1 represents Phase3 in the product lifecycle curve, which is the declining phase.

#### The regression model

According to the proposed framework, there is an initial hypothesis of data linearity. When applying the regression models on DS1, the auto.arima function from ‘pmdarima’ library is employed to automatically find the best combinations of regression model hyperparameters based on the objective function of minimum AIC, resulting in two sets of combinations: Model One or M1 using the stepwise function, and Model Two or M2 without the stepwise function. The selected models and their respective hyperparameters, along with the corresponding AIC and BIC values, are displayed in Table 3.

**Table 3:** Results of regression models hyperparameters for DS1.

Selected Model	M1 Hyperparameters	AIC	BIC	M2 Hyperparameters	AIC	BIC
SARIMA	(0,1,2) (1,0,1) ^12^	4245.2	4262.9	(2,1,0) (1,0,1) ^12^ intercept	4270.7	4292.0

**Fig. 3:**
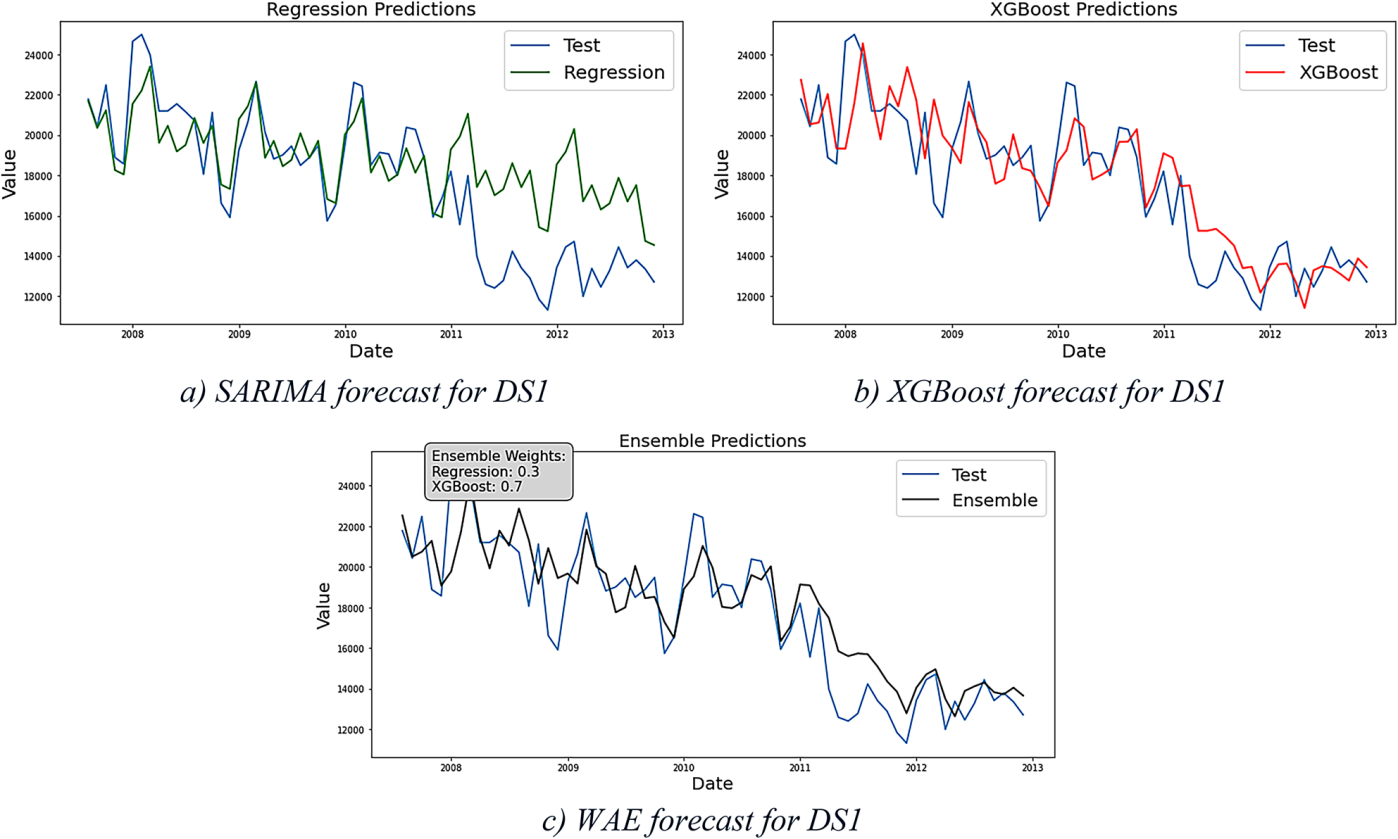
Forecast results for DS1.

Figure 3a shows the forecasts for DS1 using regression model. The figure shows only the test portion of the test, with the actual data (in blue) and the forecast (in green). The curves obtained from this step have been utilized to assess the performance of forecasting models with the datasets.

#### Diagnostics of regression model results

**Fig. 4:**
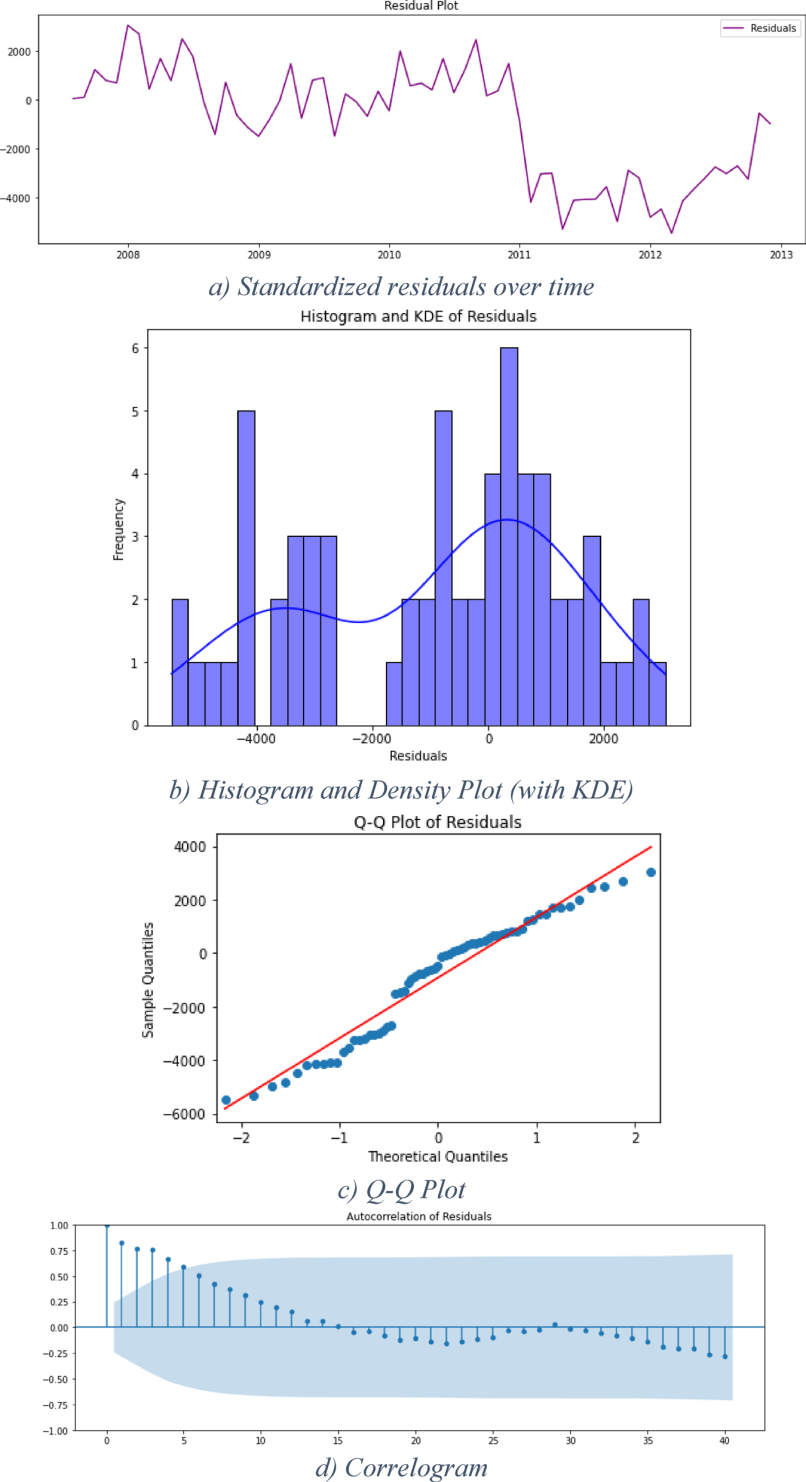
Residual analysis using plot_diagnostics for DS 1.

The results obtained by the regression model are now tested to accept or reject the initial assumption of data linearity using the “plot_diagnostics” function. Figure 4 illustrates the outcomes of this step for DS1, featuring four distinct graphs:

- Standardized residuals over time show the model couldn’t handle the patterns in the data.
- Histogram plus estimated density of standardized residuals, with a Normal (0,1) density plotted for reference. As the two plots were not identical, then there is a pattern in the residuals.
- Normal Q-Q plot, with Normal reference line: all the blue dots should fall perfectly in line with the red line for no pattern in residuals. The graph suggests a skewed distribution.
- Correlogram (i.e., ACF plot): displays a correlation at lag 5. It implies that there is some pattern in the residual errors which are not explained by the regression model.

*From* the interpretation of the graphs, the residual errors have a pattern, and this indicates nonlinearity in the data set. Hence, XGBoost model will be used to handle nonlinear data.

#### The XGBoost forecasting model

**Fig. 5:**
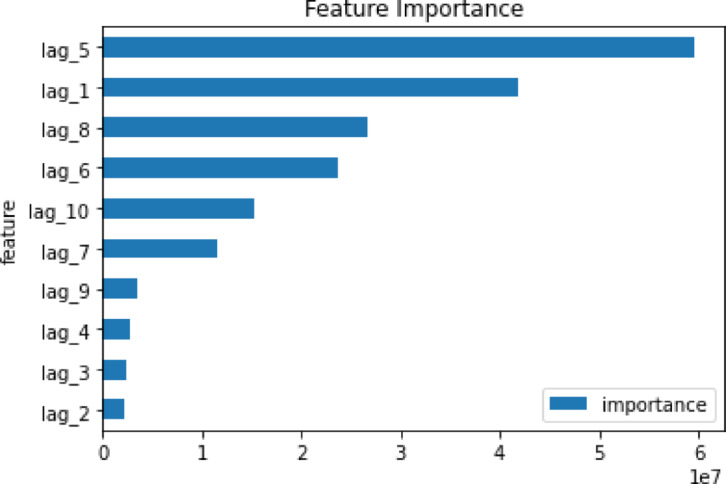
Feature importance for DS1.

In this study, the F-score of each lag was observed, as depicted in Figure 5. The graph considers both the frequency of a feature’s appearance in tree nodes and the average gain—or improvement in model performance—contributed by splits involving that feature. Lag 5 represents the most important feature, while other lags, such as lag 1, exhibit a high F-score value, indicating a strong time dependency within the data.

The results obtained from the XGBoost model following the previously conducted hyperparameter tuning step are displayed in Figure 3b. The curve illustrates the proposed XGBoost model’s capability to handle declining data and overcome the level change observed in the test set.

#### The WAE model

Despite the visual comparison indicating the superiority of the XGBoost model over the SARIMA model for DS1, the framework proceeds with its subsequent steps to generate forecasts using the WAE model. As identified earlier during the model diagnostic stage, the analysis revealed the presence of mixed patterns in the data for DS1, encompassing both linearity and nonlinearity. The WAE model is specifically crafted to enhance performance when dealing with such diverse data patterns. The WAE model assigned 0.3 and 0.7 weights for SARIMA and XGBoost models respectively. Forecast results are shown in Fig. 3c (in black). The figure shows a competitive result achieved by the WAE model. Notably, the weight of XGBoost is larger than that of the SARIMA model, influenced by the level change in the test set.

#### Models evaluation

Performance measures for the three models are calculated and summarized in Table 4. A comparison of these models reveals that the developed WAE model, with a weight distribution of 0.3 for SARIMA and 0.7 for XGBoost, exhibits a remarkable ability to forecast the the decline phase of the product life cycle. This is followed by the XGBoost model, with SARIMA trailing as the least performing model.

**Table 4:** Models evaluation results for DS1.

DS1	MSE	RMSE	MAE	MAPE (%)
SARIMA	6,980,822	2642	2019	14
XGBoost	3,307,421	1818	1400	8
WAE	2,721,807	1649	1325	8

### Framework results for DS2

**Fig. 6:**
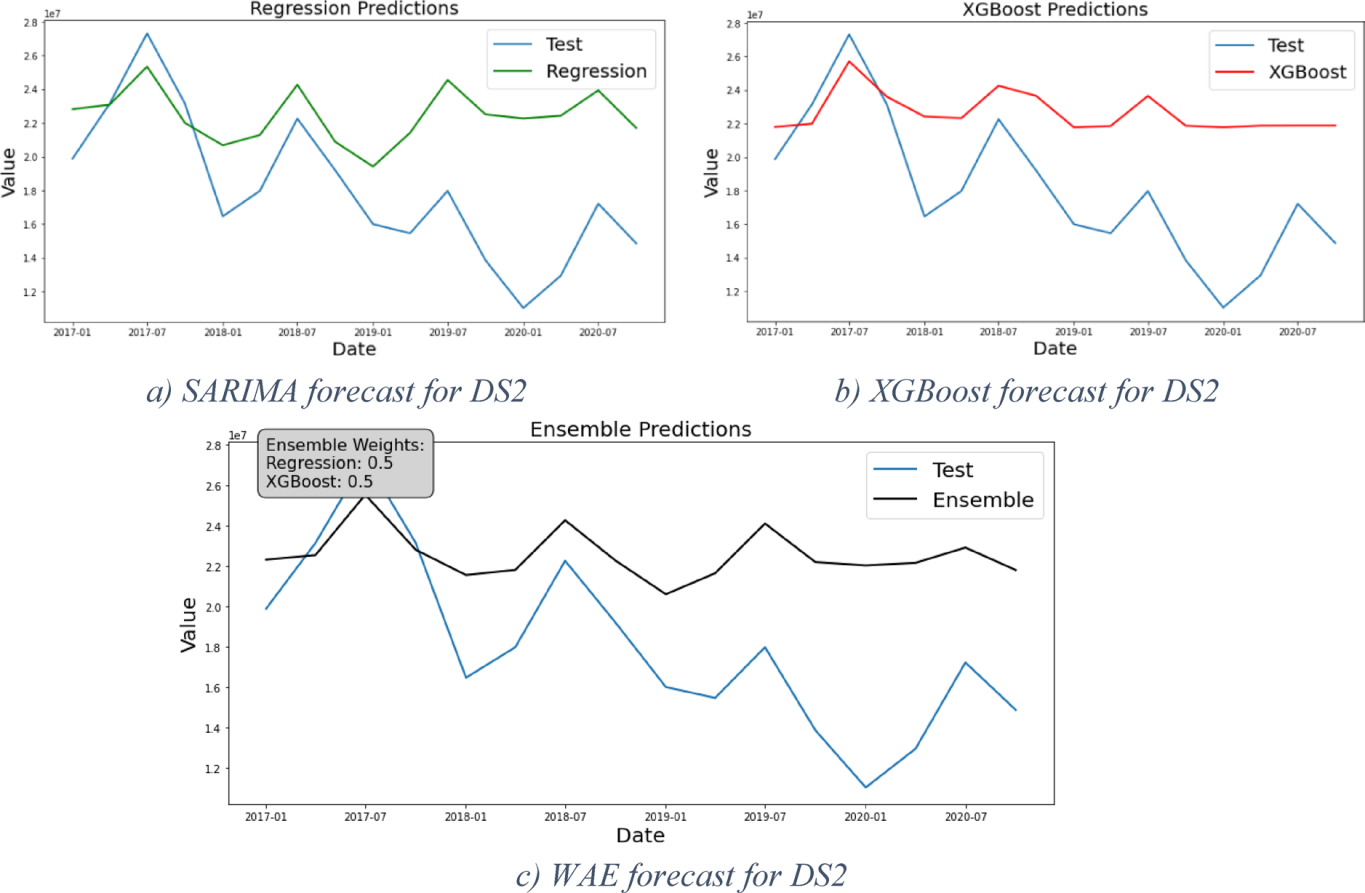
Forecast results for DS2.

DS2 resembles the early stage of Phase3, the start of the declining stage in the product life cycle. As shown in Table 2, DS2 has a seasonal pattern. The SARIMA model is used for forecasting. Figure 6a shows the forecast (in green) versus the test portion of the dataset (in red). The diagnostic stage reveals a pattern in residuals, prompting the framework to proceed with generating forecasts using the XGBoost model shown in Fig. 6b. It is noticed that the results of both models are close. Based on the grid search for the weights of the WAE model, equal weights were given to both models, and the forecast is in Fig. 6c (in black).

Table 5 further demonstrates that the WAE model exhibits the best performance, with the SARIMA model closely trailing due to the limited impact of nonlinearity in this case. However, However, DS2 was used for the sake of testing the framework performance under smaller datasets—80 observations—and shows relatively high error. Hence, DS2 needs further investigation to study the effect of the sample size of the datasets on the results as it requires other models to handle it.

**Table 5:** Models evaluation results for DS2.

	MSE	RMSE	MAE	MAPE (%)
Regressive	32,665,469,191,440	5,715,371	4,762,220	32
XGBoost	32,705,637,806,908	5,718,884	4,945,515	33
WAE	32,241,403,606,754	5,678,151	4,825,182	32

#### Framework results for DS3

**Fig. 7:**
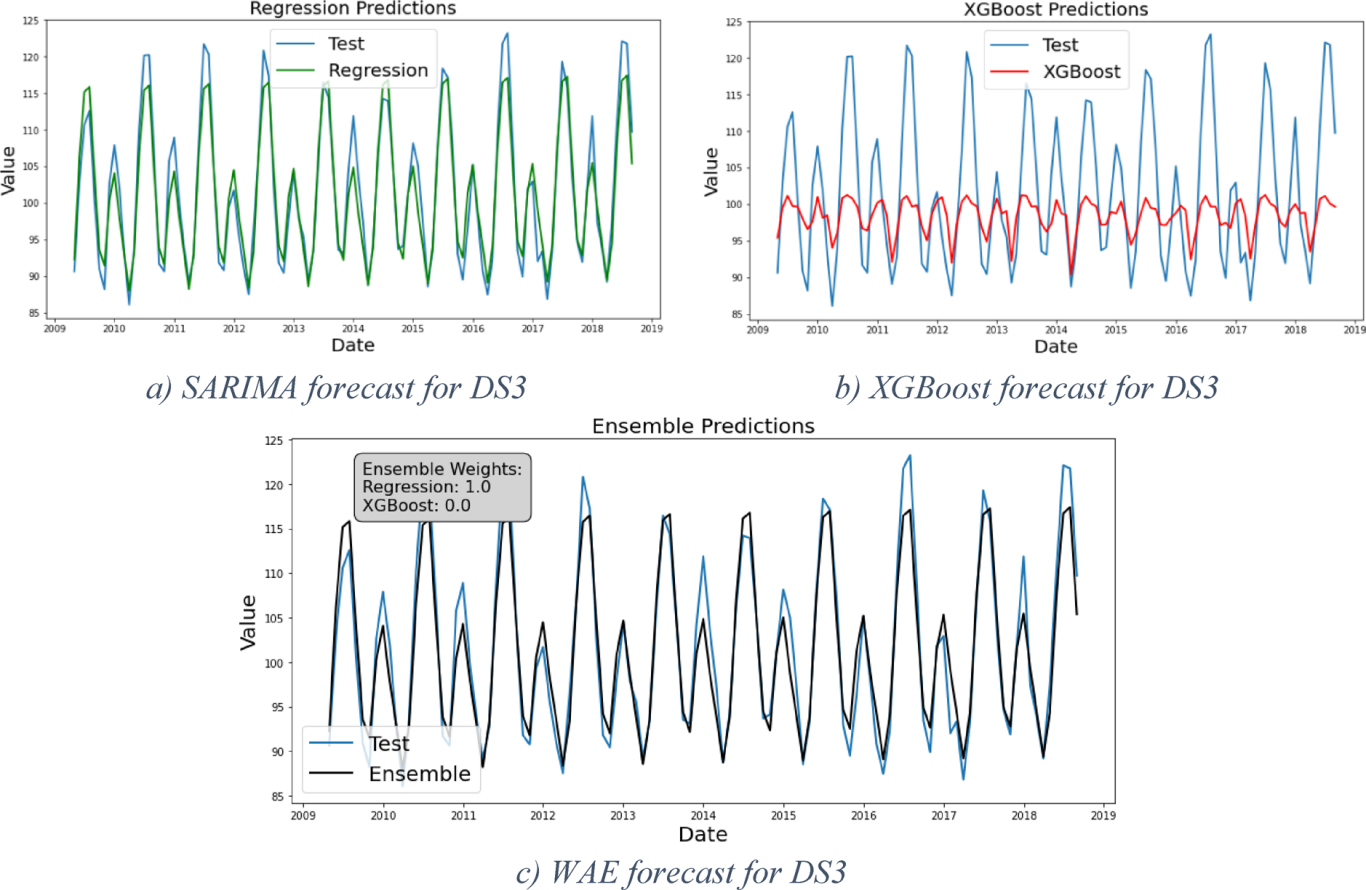
Forecast results forDS3.

DS3 represents Phase1, wherein the curve progresses from the introduction stage through growth and ultimately reaches maturity, with the target of the forecast being the maturity phase. Applying the SARIMA model resulted in the forecasts shown in Fig. 7a (in green). The residual analysis reveals no clear pattern in the data, confirming the linearity assumption. While the framework stops at this stage, we proceed with the following stages of the framework for validation reasons. The framework should not give forecasts using the XGBoost (in red) and the WAE model (in black), however, the results are given in Fig. 7. The grid search process for the WAE model determines the optimal weights as 100% for SARIMA and 0% for XGBoost. This matches our hypothesis that the data sets with linear pattern are best solved using the regression models, and that the machine learning model is not promising for the linear data. These results proved to be the best given the values in Table 6, confirming that SARIMA is the best candidate for DS3.

**Table 6:** Models evaluation results for DS3.

DS3	MSE	RMSE	MAE	MAPE (%)
Regressive	8.23	2.87	2.28	2
XGBoost	173.97	13.19	10.23	9
WAE	8.23	2.87	2.28	2

### Framework results for DS4 and DS5

**Fig. 8:**
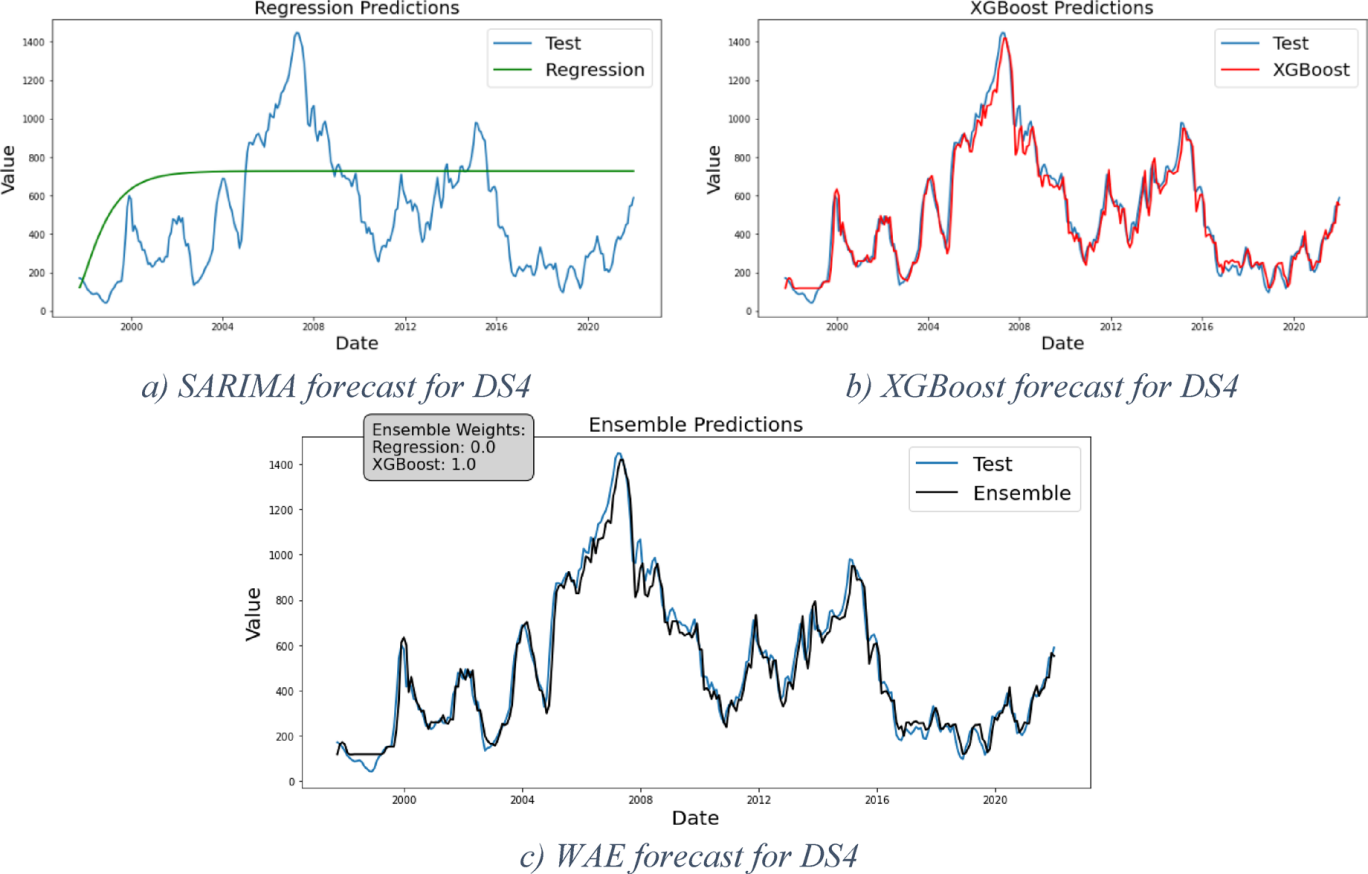
Forecast results for DS4.

DS4 and DS5 are two cases for erratic demand patterns. DS4 represents another instance of Phase2 that exclusively focuses on portraying the maturity stage, while DS5 represents Phase1 or the end of the introduction stage where the curve starts to take off. DS5 presents an additional challenge due to a significant shift in demand levels toward the end of the period under study. After obtaining results from the regression models—displayed in Fig. 8a for DS4 and 9.a for DS5 (both in green)—residual analysis reveals the presence of nonlinearity in the data suggesting the necessity to proceed further in the framework stages.

**Fig. 9:**
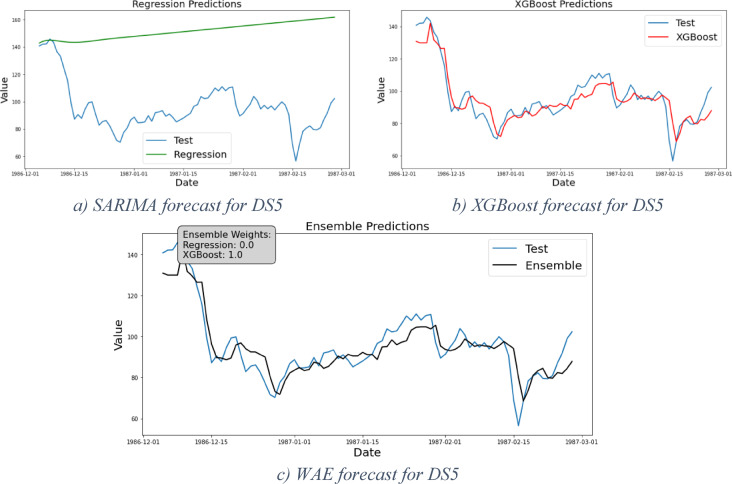
Forecast results for DS5.

The XGBoost forecast clearly demonstrates the model’s strong predictive capability, as illustrated in Figs. 8b and 9b (shown in red). In these cases, the WAE model assigns a weight of 0 to the regression models and 1 to the XGBoost model. As expected, the evaluation results presented in Table 7 confirm that XGBoost delivers the best performance for DS4, with Table 8 showing the same outcome for DS5.

**Table 7:** Models evaluation results for DS4.

DS4	MSE	RMSE	MAE	MAPE (%)
Regressive	122,274	349.6	302.06	118
XGBoost	3549	59.57	44.39	13
WAE	3549	59.57	44.39	13

**Table 8:** Models evaluation results for DS5.

DS5	MSE	RMSE	MAE	MAPE (%)
Regressive	3510.13	59.25	55.81	63
XGBoost	54.57	7.39	5.65	6
WAE	54.57	7.39	5.65	6

## Conclusion and future work

This research presents a novel, adaptive framework for forecasting general time series data, designed to accommodate various demand patterns commonly encountered across different stages of the product life cycle (PLC). The framework integrates classical regression methods from the ARIMA family with the machine learning capabilities of XGBoost through a Weighted Average Ensemble (WAE) model. This hybrid approach enhances forecasting flexibility and accuracy, particularly when applied to datasets with mixed linear and nonlinear characteristics. The methodology assumes linearity at the outset, applying an appropriate ARIMA-based model for initial forecasting. Residual diagnostics are then used to evaluate the adequacy of the linear assumption. If nonlinearity is detected, the XGBoost model is activated, and the final forecast is generated using the WAE model. The ensemble weights are optimized through a grid search mechanism that minimizes RMSE, allowing the model to dynamically prioritize the most effective method based on the data’s underlying behavior. Validation across five real-world datasets demonstrates the framework’s adaptability. In mixed-pattern datasets (DS1 and DS2), the WAE model achieved best performance by balancing the strengths of ARIMA and XGBoost, with different weight configurations depending on the presence of linear or seasonal trends. In DS3, the residual analysis supported the sufficiency of SARIMA, while in DS4, the nonlinearity was evident leading the WAE model to assign full weight to XGBoost, significantly improving forecast accuracy. Overall, the results show that the proposed framework consistently outperforms standalone models when applied to diverse PLC stages, including growth, maturity, and especially the underexplored declining phase. The ensemble approach capitalizes on the interpretability and pattern recognition strengths of ARIMA models, alongside the nonlinear learning capacity of XGBoost, resulting in a forecasting system that offers improved accuracy, reduced bias, adaptability to changing data behaviors, and resilience against overfitting. In addition to its forecasting performance, the framework is practical for industrial applications involving high-volume, continuous, or erratic demand patterns. Sectors such as home appliances, automotive manufacturing, pharmaceuticals, and food production can benefit from its ability to simulate and forecast across different PLC scenarios—providing a strategic tool for inventory planning and product expansion strategies.

Future improvements to enhance the proposed framework include integrating deep learning models like LSTM for better long-term forecasting and adapting it to handle intermittent demand using methods such as Croston’s model. Another direction is to use metaheuristic algorithms for ensemble weight optimization. Full automation of the framework is recommended to support real-time deployment. Additionally, evaluating the effect of sample size on performance will help assess its robustness in low-data scenarios.

## Data Availability

The data sets used and analyzed during the study are publicly available and can also be shared upon request.

## Abbreviations

ACF: Autocorrelation function
MLA: Machine learning algorithm
ADF: Augmented Dickey Fuller
MSE: Mean square error
AIC: Akaike information criterion
PACF: Partial autocorrelation function
AR: Autoregression
RMSE: Root mean square error
ARMA: Autoregression moving average
SARIMA: Seasonal autoregression integrated moving average
ARIMA: Autoregression integrated moving average
SD: Seasonal decomposition
BIC: Bayesian information criterion
SVM: Support vector machine
FFT: Fast Fourier transformation
TSMs: Time series methods
KDE: Kernel density estimate
XGBoost: Extreme gradient boosting
MAE: Mean absolute error
WAE: Weighted average ensemble
MAPE: Mean absolute percentage error
